# Innovative Multiplex PCR Assay for Detection of *tlh*, *trh*, and *tdh* Genes in *Vibrio parahaemolyticus* with Reference to the U.S. FDA’s *Bacteriological Analytical Manual* (*BAM*)

**DOI:** 10.3390/pathogens13090774

**Published:** 2024-09-07

**Authors:** Seong Bin Park, Yan Zhang

**Affiliations:** Experimental Seafood Processing Laboratory, Coastal Research & Extension Center, Mississippi State University, Pascagoula, MS 39567, USA

**Keywords:** improved multiplex PCR, U.S. FDA *BAM*, tlh, trh, tdh, vibrio parahaemolyticus

## Abstract

*Vibrio parahaemolyticus* is an important foodborne bacterium that causes severe gastroenteritis following the consumption of contaminated seafood. To identify *V. parahaemolyticus* and determine its pathogenicity, the U.S. Food and Drug Administration (FDA)’s *Bacteriological Analytical Manual* (*BAM*) recommends a multiplex polymerase chain reaction (PCR) protocol to simultaneously detect the species-specific thermolabile hemolysin (*tlh*) gene and the pathogenic thermostable-related hemolysin (*trh*) and thermostable-direct hemolysin (*tdh)* genes. However, this assay has shown two limitations: difficulty in separating the amplicons of the *trh* (486 bp) and *tlh* (450 bp) genes due to their highly similar sizes, and the weaker band exhibited by the *tdh* gene amplicon (270 bp). The present study aimed to improve the *BAM*’s multiplex PCR assay by separating the three amplicons with similar intensity. A new primer set was applied for the *tlh* gene (369 bp) alongside the existing primers for the *trh* and *tdh* genes. The amplicons for the three genes were effectively separated by electrophoresis on a 2% tris-borate-EDTA (TBE) agarose gel within 45 min. Primer concentrations of 0.25 µM for three genes produced a significant amount of amplicons among various combinations of primer concentrations with 35 PCR cycles. This assay exhibited a detection limit of 10 pg of bacterial DNA, demonstrating its high sensitivity. It did not display amplicons from nine *Vibrio* species known to be human pathogens or from 18 well-documented foodborne pathogens. Therefore, the present multiplex PCR protocol could help overcome the limitations of existing assays and provide a more reliable method for detecting the three genes of *V. parahaemolyticus*.

## 1. Introduction

*Vibrio parahaemolyticus* poses a significant threat to public health through the consumption of contaminated seafood [1]. According to the U.S. Centers for Disease Control and Prevention (CDC), approximately 84,000 people suffer from Vibrio-related illnesses annually [2,3]. This Gram-negative halophilic bacterium inhabits estuarine and marine environments and can naturally infiltrate oysters [2,3,4]. However, the number of bacteria significantly increases in oysters during the warm-water season, and improper distribution or handling can further accelerate bacterial contamination, leading to public health concerns [5,6]. For instance, the Pacific *V. parahaemolyticus* strain (O4 serotype/sequence type 36) severely impacted Oyster Bay, NY, causing a significant increase in reported illnesses and extended closures in Long Island Sound, including major areas in Connecticut [7]. This outbreak was linked to the consumption of shellfish harvested from Oyster Bay Harbor, New York, between April and August 2012. By 2013, the outbreak with the same indistinguishable strain had spread from Virginia to Massachusetts, causing over 100 reported illnesses in 13 states and resulting in unprecedented closures and recalls [8]. This crisis made the industry view the situation as an existential threat and become more open to implementing controls to restore their critical summer operations [9].

A multiplex polymerase chain reaction (PCR) protocol is listed as one of the standard methods in the U.S. Food and Drug Administration (FDA)’s *Bacteriological Analytical Manual* (*BAM*) for identifying *V. parahaemolyticus* and determining its pathogenicity simultaneously, while information for the real-time PCR assay is still not available [10]. This assay is designed to yield three bands showing the thermolabile hemolysin (*tlh*) gene as a unique identification marker and the thermostable-related hemolysin (*trh*) and thermostable-direct hemolysin (*tdh*) genes as pathogenic markers of *V. parahaemolyticus*. Studies have demonstrated that all examined *V. parahaemolyticus* showed the amplification of *tlh* gene, with no positive results in closely related *Vibrio* spp. and other foodborne pathogens [11,12,13]. Moreover, clinical investigations have indicated that *V. parahaemolyticus* isolated from human patients carry the *trh* and *tdh* genes, which are potentially responsible for seafood-related illnesses and deaths [14,15,16]. Therefore, identifying *V. parahaemolyticus* and determining its pathogenicity in oysters are crucial steps to prevent foodborne illnesses and protect the domestic seafood industry.

Recently, we conducted the multiplex PCR recommended by *BAM* to identify *V. parahaemolyticus* and assessed its pathogenicity in oysters from the U.S. Gulf Coast. After 25 PCR cycles using a positive control strain (F11-3A), the amplicons were loaded onto a 1.5% tris-borate-EDTA (TBE) agarose gel [2,11]. However, the three bands did not appear even after 90 min of electrophoresis. The bands for *trh* (486 bp) and *tlh* (450 bp) were not separated well, and the *tdh* band (270 bp) was weaker compared to others. This method was originally developed by Bej et al., and the major difference between the multiplex PCR method of *BAM* and Bej et al. was the number of PCR cycles (25 cycles of *BAM* and 30 cycles of Bej et al.) [11]. We found that the original method produced a thicker *tdh* band than *BAM*, but the bands for *trh* and *tlh* were not well separated even after 90 min of electrophoresis.

In the present study, we aimed to improve the multiplex PCR method recommended by *BAM* by modifying the primers to achieve efficient separation of the three target genes and enhance the amplification of the *tdh* gene. Additionally, we examined the relative concentrations of primers, PCR cycling conditions, amounts of template DNA, the percentage of agarose, and electrophoresis time to produce three even amplicons, thereby confirming the specificity of the three sets of primers. The detection limit of the assay was evaluated using various concentrations of *V. parahaemolyticus* DNA, and the specificity of the assay was tested using nine *Vibrio* strains known to be human pathogens and 18 well-documented foodborne pathogens.

## 2. Materials and Methods

### 2.1. Bacteria, Genomic DNA, and Primers

*Vibrio parahaemolyticus* F11-3A was used as the reference strain to amplify *tlh*, *trh,* and *tdh* genes [2,11]. Other Vibrio and foodborne bacteria were used to determine the specificity of the multiplex PCR, including *V. parahaemolyticus* ATCC 17802, *V. parahaemolyticus* ATCC 35118, *V. vulnificus* ATCC 33147, *V. vulnificus* ATCC 27562, *V. vulnificus* ATCC 33815, *V. metschnikovii*, *V. fluvialis* ATCC 33809, *V. mimicus* ATCC 33655, *V. furnissii* ATCC 35627, *V. cholerae* ATCC 39315, *V. alginolyticus* ATCC 33840, *Escherichia coli* ATCC 51739, *E. coli* K-12, *E. coli* O157:H7 ATCC 43895, *Listeria monocytogenes* F5069, *Lactobacillus buchneri* ATCC 12936, *Listeria innocua* ATCC 33090, *Salmonella enterica* Serovar Typhimurium 14028, *S. enterica* Serovar Gaminara F2712, *S. enterica* Serovar Montevideo ATCC BAA-1735, *S. enterica* Serovar Senftenburg ATCC 43845, *S. enterica* Serovar Enteritidis E190-88, *S. enterica* Serovar Choleraesuis ATCC 10708, *Bacillus subtilis* ATCC 9372, *Clostridium perfringens* ATCC 13124, *Enterococcus faecalis* ATCC 344, *Lactobacillus acidophilus* NRRL B1910, and *Staphylococcus aureus* ATCC 25923, *Shigella flexineri* ATCC 12022. All bacteria were cultured on tryptic soy agar (TSA, Remel, San Diego, CA, USA) or in tryptic soy broth (TSB, Remel) at 37 °C. Bacterial DNA samples were extracted using the Quick-DNA Fungal/Bacterial Miniprep Kit (Zymo Research, Irvine, CA, USA) according to the manufacturer’s instructions and stored at −80 °C until use. The concentration of bacterial DNA was measured using the NanoDrop spectrophotometer (Thermo Fisher Scientific, Waltham, MA, USA) by measuring the absorbance at 260 nm. Primers listed in Table 1 were used for multiplex PCR. The *tlh* gene primers were designed using SnapGene software (version 5.2, San Diego, CA, USA) targeting a specific region within the gene (gene ID: GU971655.1, MH047289.1, OP270227.1, accessed on 15 January 2024). Primers for the amplification of *trh* and *tdh* genes were adopted from protocols described by both Bej et al. and *BAM* [2,11] (Table 1).

### 2.2. Multiplex PCR Condition

Three multiplex PCR methods were employed. The PCR mixture (50 µL) of both *BAM* and Bej et al. [11] consisted of the bacterial DNA (10 ng to 10 pg) from *V. parahaemolyticus* F11-3A, 1 µM of each of the primers (5 µL of each primer from 10 µM stock), 5 µL of a 10 X PCR buffer, 320 µM of each of the dNTPs (8 µL of a 8 mM stock dNTPs), 2.5 units of Dream Taq Green DNA polymerase (0.5 µL of 5 units/µL, Thermo Scientific, Vilnius, Lithuania), and 5.5 µL of water. The amplification condition of *BAM* was 1 cycle at 94 °C for 3 min, followed by 25 cycles of 94 °C for 1 min, 60 °C for 1 min, and 72 °C for 2 min, with a final extension at 72 °C for 3 min, while the amplification condition of Bej et al. [11] was 1 cycle at 94 °C for 3 min, followed by 30 cycles of 94 °C for 1 min, 58 °C for 1 min, and 72 °C for 1 min, with a final extension at 72 °C for 5 min.

Our PCR mixture was composed of the bacterial DNA (1 ng to 1 pg), 0.25 µM of each of the primers (1.25 µL of each primer from 10 µM stock), 5 µL of a 10 X PCR buffer, 320 µM of each of the dNTPs (8 µL of a 8 mM stock dNTPs), 1.5 units of Dream Taq Green DNA polymerase (0.3 µL of 5 units/µL, Thermo Scientific, Vilnius, Lithuania), and 28.2 µL of water. The amplification condition for this study was 1 cycle at 94 °C for 3 min, followed by 35 cycles of 94 °C for 1 min, 58 °C for 1 min, and 72 °C for 1 min, with a final extension at 72 °C for 5 min.

Electrophoresis was conducted using 1.5% TBE (TBE, Alfa Aesar, Ward Hill, MA, USA) agarose gels containing the SYBR Safe DNA gel stain (Invitrogen, Waltham, MA, USA) for 90 min to optimize band separation. Similarly, electrophoresis through 2% TBE gel was run for 45 min. The gel was visualized using the Gel Doc XR+ system (Bio-Rad, Hercules, CA, USA).

### 2.3. Optimization, Sensitivity, and Specificity of Multiplex PCR Assay

To determine the optimal PCR cycles for our multiplex PCR, the amplification condition was examined for 1 cycle at 94 °C for 3 min, followed by 30 or 35 cycles of 94 °C for 1 min, 58 °C for 1 min, and 72 °C for 1 min, with a final extension at 72 °C for 5 min. Various concentrations (1 ng, 100 pg, 10 pg, 1 pg, and 100 fg) of *V. parahaemolyticus* F11-3A DNA were used to determine the sensitivity of the multiplex PCR. After genomic DNA was extracted from other *Vibrio* strains and foodborne pathogenic bacteria, 1 ng of each of the bacterial DNA samples was employed to determine the specificity of the multiplex PCR.

## 3. Results and Discussion

### 3.1. Multiplex PCR Assay of BAM and Bej et al. [11]

**Figure 1:**
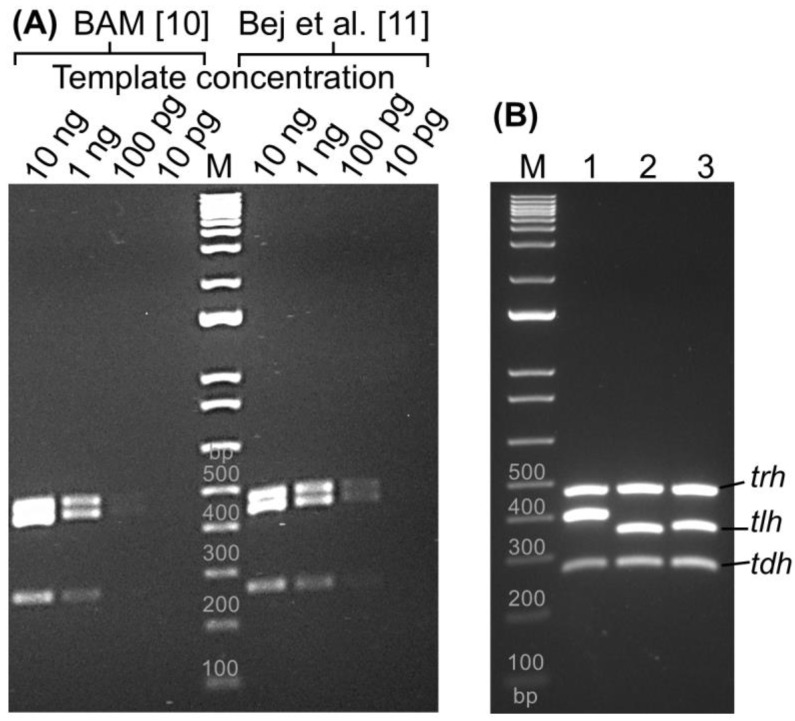
**Multiplex PCR analysis of *Vibrio parahaemolyticus tlh*, *trh,* and *tdh* genes.** The protocols were based on the *Bacteriological Analytical Manual* (*BAM*) [10] of the U.S. FDA and a study by Bej et al. [11] (Panel **A**). (Panel **B**) showed the current multiplex PCR with various combinations of primers to amplify the middle band of *tlh* gene (1: 403 bp, 2: 359 bp, and 3: 369 bp). M: Molecular weight marker. The electrophoreses were run for 90 min to separate three bands through a 1.5% TBE agarose gel.

Multiplex PCR assays were conducted based on the method described by Bej et al. and *BAM* to evaluate the simultaneous detection of *tlh*, *trh*, and *tdh* genes of *V. parahaemolyticus* [10,11]. Since the *BAM* protocol originated from Bej et al. [11], both protocols shared identical reaction components, including the PCR mixture and primer sequences, except for modifications to the cycling conditions in the *BAM* protocol. The sensitivity of the assay was assessed using different concentrations of F11-3A DNA with all three primer sets (Figure 1A).

The multiplex PCR assay of *BAM* and Bej et al. [11] exhibited insufficient separation of *trh* and *tlh* gene amplicons at 30 and 60 min of electrophoresis. While separation of both genes was achieved at 90 min, the bands remained unresolved with 10 ng of bacterial DNA (Figure 1A). Three genes displayed faint bands in the sample containing 100 pg of bacterial DNA and became visible at 1 ng of DNA at 90 min of electrophoresis. Therefore, the limit of detection (LOD) of the *BAM* multiplex PCR was determined to be 1 ng of genomic DNA. The multiplex PCR assay of Bej et al. [11] showed stronger band intensity for all three genes compared to *BAM* (Figure 1A). This difference was probably due to the increased number of PCR cycles employed by Bej et al. (30 cycles) compared to *BAM* (25 cycles). The LOD of this method was determined to be 100 pg of bacterial DNA.

Both multiplex PCR assays exhibited limitations in separating the amplicons of the *trh* (486 bp) and *tlh* (450 bp) genes due to their highly similar sizes. This small size difference makes it difficult to distinguish the two amplicons on a gel. Additionally, the *tdh* gene amplicon (270 bp) often displays weaker band intensity compared to *trh* and *tlh*, potentially hindering its detection. To address these limitations, our multiplex PCR was designed to effectively separate the amplicons of *trh* and *tlh* genes, allowing for clear identification of both targets. Furthermore, the assay would be optimized to generate amplicons for all three genes (*trh*, *tlh*, and *tdh*) with similar band intensities, facilitating easier detection and analysis. This improved design aimed to overcome the limitations of the existing assays and provide a more reliable method for detecting these genes.

### 3.2. Optimization of the Current Multiplex PCR

To optimize a multiplex PCR assay for efficient separation and detection of three genes (*tlh*, *trh*, and *tdh*) in *V. parahaemolyticus*, three primer sets for *tlh* gene were examined for our enhanced multiplex PCR (Table 1). The primers for amplification of *trh* (486 bp) and *tdh* (270 bp) were adopted from *BAM* and Bej et al. [10,11]. The middle bands (*tlh*) of lane 1, 2, and 3 in Figure 1B were amplified using the combination of VP_TLH_L and VP_TLH_R2 (403 bp), VP_TLH_F2 and VP_TLH_R (359 bp), and VP_TLH_F2 and VP_TLH_R2 (369 bp), respectively. All three candidates for *tlh* gene displayed specific amplicons along with the expected amplicons for *trh* (486 bp) and *tdh* (252 bp) genes on the gel, with no non-specific products observed. Notably, lane 3 using the VP_TLH_F2/VP_TLH_R2 primers displayed the most consistent separation between all three target bands. Therefore, this primer set was selected for the optimized multiplex PCR.

**Figure 2:**
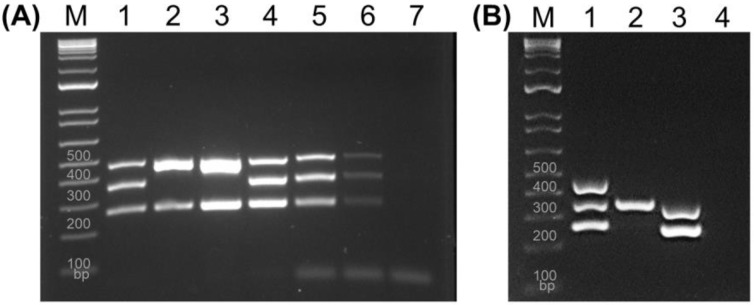
Validation of PCR cycle number, sensitivity, and specificity of the current multiplex PCR. (Panel **A**) The multiplex PCRs were conducted with 30 cycles (Lane 1 and 2) and 35 cycles (Lane 3 to 7) using the protocol of Bej et al. [11] (Lane 2 and 3) and the current multiplex PCR (Lane 1, 4, 5, 6, and 7). The sensitivity of the current multiplex PCR was determined using various DNA concentrations (lane 4: 1ng, lane 5: 100 pg, lane 6: 10 pg, and lane 7: 1 pg). (Panel **B**) Specificity of the current multiplex PCR. *Vibrio parahaemolyticus* F11-3A exhibited positive results for the *trh*, *tlh*, and *tdh* genes (lane 1). *V. parahaemolyticus* ATCC 17802 was positive only for the *tlh* gene (lane 2). *V. parahaemolyticus* ATCC 35118 showed positivity for the *tlh* and *tdh* genes (lane 3). None of the other tested *Vibrio* strains and foodborne pathogenic bacteria displayed amplification of three genes (lane 4).

Our results, similar to those of previously reported methods (*BAM* and Bej et al.), showed a weaker band intensity for the *tdh* gene (Figure 1B) compared to the *trh* and *tlh* genes at longer electrophoresis times (90 min). When uneven amplification occurs, it is important to adjust the proportions of the primers in the reaction mixture and optimize the cycling conditions [17]. To enhance band intensity for the *tdh* gene, five different primer concentrations were tested. A concentration of 0.25 µM for three primers yielded three amplicons with significant and uniform band intensity at shorter electrophoresis times—45 min on a 2% gel (Lane 1 in Figure 2A). In contrast, the Bej et al. multiplex PCR (Lane 2 and 3 in Figure 2A) failed to separate *trh* and *tlh* genes. Additionally, 35 cycles of PCR (Lane 4 in Figure 2A) produced stronger band intensities in three genes compared to 30 cycles of PCR (Lane 1 in Figure 2A). Therefore, our multiplex PCR significantly improved the amplicon intensity using 0.25 µM of primers, 35 cycles of PCR, and electrophoresis on 2% TBE gel for 45 min.

### 3.3. Sensitivity and Specificity of Our Multiplex PCR

A previous study reported a multiplex PCR for detection of *groEL*, *trh*, and *tdh* genes with a limit of detection (LOD) of 200 pg of *V. parahaemolyticus* DNA using 30 PCR cycles [18]. Another study demonstrated that the LOD of foodborne bacterial DNA in their multiplex PCR assay was 6.4 pg for *Staphylococcus aureus*, 32 pg for *Escherichia coli* O157:H7, 800 pg for *Listeria monocytogenes*, 160 pg for *Shigella flexneri*, and 32 pg for *Salmonella enterica* serovar Enteritidis using 35 cycles [19]. In this study, the sensitivity of our multiplex PCR assay was determined using various concentrations of F11-3A DNA (Lane 4, 5, 6, and 7 in Figure 2A). The overall LOD for all three genes was 10 pg of bacterial DNA. This sensitivity was 100 times higher than the results obtained using the *BAM* method (Figure 1A).

The specificity of the multiplex PCR assay was evaluated using three *V. parahaemolyticus* (lanes 1–3 in Figure 2B), nine *Vibrio* species known to be human pathogens, and 18 well-documented foodborne pathogens [10,20,21,22]. All three target genes were successfully amplified using F11-3A (Lane 1). In contrast, *V. parahaemolyticus* ATCC 17802 (Lane 2) showed only *tlh*-positive results, while *V. parahaemolyticus* ATCC 35118 (Lane 3) displayed both *tlh-* and *tdh*-positive results, consistent with previous studies [11,13]. Notably, the template DNA of all the other bacteria yielded the same identical product (none), as shown in lane 4, demonstrating high specificity. Therefore, this assay can be considered a viable alternative multiplex PCR with reference to the *BAM* protocol.

## 4. Conclusions

This study presents an efficient multiplex PCR assay for the detection of *tlh*, *trh*, and *tdh* genes of *V. parahaemolyticus*. To enhance the multiplex PCR recommended by *BAM*, we redesigned the primer set, optimized the concentration of primers, and adjusted the conditions of PCR cycles and gel electrophoresis. Our assay effectively separated the amplicons of three genes with similarly clear band intensities, facilitating their detection. Given its high sensitivity and specificity, we believe this method could be highly beneficial for researchers eager to detect the three genes simultaneously using the *BAM* method with slight modifications.

## Figures and Tables

**Table 1:** Primers for the amplification of *tlh*, *trh*, and *tdh* genes using the multiplex PCR.

Names	Genes	Sequences (5′-3′)	Size (bp)	References
VP_TLH_L	*tlh*	AAAGCGGATTATGCAGAAGCACTG	450	[11]
VP_TLH_R		GCTACTTTCTAGCATTTTCTCTGC		
VP_TRH_L	*trh*	TTGGCTTCGATATTTTCAGTATCT	486	
VP_TRH_R		CATAACAAACATATGCCCATTTCCG		
VP_TDH_L	*tdh*	GTAAAGGTCTCTGACTTTTGGAC	270	
VP_TDH_R		TGGAATAGAACCTTCATCTTCACC		
VP_TLH_F2	*tlh*	CTCAGTTTAAGTACTCAACACAAGAAGAGAT	369	This study and [11]
VP_TLH_R2		CTAAGTTGTTGCTACTTTCTAGCATTTTCT		
VP_TLH_F2	*tlh*	CTCAGTTTAAGTACTCAACACAAGAAGAGAT	359	
VP_TLH_R		GCTACTTTCTAGCATTTTCTCTGC		
VP_TLH_L	*tlh*	AAAGCGGATTATGCAGAAGCACTG	403	
VP_TLH_R2		CTAAGTTGTTGCTACTTTCTAGCATTTTCT		

## Data Availability

Data are contained within the article.
